# *ARABIDOPSIS HOMOLOG of TRITHORAX1* (*ATX1*) is required for cell production, patterning, and morphogenesis in root development

**DOI:** 10.1093/jxb/eru355

**Published:** 2014-09-09

**Authors:** Selene Napsucialy-Mendivil, Raúl Alvarez-Venegas, Svetlana Shishkova, Joseph G. Dubrovsky

**Affiliations:** ^1^Departamento de Biología Molecular de Plantas, Instituto de Biotecnología, Universidad Nacional Autónoma de México (UNAM), Apartado Postal 510–3, 62250 Cuernavaca, Morelos, México; ^2^Departamento de Ingeniería Genética, Centro de Investigación y de Estudios Avanzados, Unidad Irapuato, Irapuato, Gto., CP 36821, México

**Keywords:** *Arabidopsis*, lateral root development, patterning, root apical meristem, root development, root morphogenesis, root system.

## Abstract

*ARABIDOPSIS HOMOLOG of TRITHORAX1* (*ATX1/SDG27*), a known regulator of flower development, encodes a H3K4histone methyltransferase that maintains a number of genes in an active state. In this study, the role of ATX1 in root development was evaluated. The loss-of-function mutant *atx1-1* was impaired in primary root growth. The data suggest that *ATX1* controls root growth by regulating cell cycle duration, cell production, and the transition from cell proliferation in the root apical meristem (RAM) to cell elongation. In *atx1-1*, the quiescent centre (QC) cells were irregular in shape and more expanded than those of the wild type. This feature, together with the atypical distribution of T-divisions, the presence of oblique divisions, and the abnormal cell patterning in the RAM, suggests a lack of coordination between cell division and cell growth in the mutant. The expression domain of QC-specific markers was expanded both in the primary RAM and in the developing lateral root primordia of *atx1-1* plants. These abnormalities were independent of auxin-response gradients. *ATX1* was also found to be required for lateral root initiation, morphogenesis, and emergence. The time from lateral root initiation to emergence was significantly extended in the *atx1-1* mutant. Overall, these data suggest that *ATX1* is involved in the timing of root development, stem cell niche maintenance, and cell patterning during primary and lateral root development. Thus, *ATX1* emerges as an important player in root system architecture.

## Summary

Methyltransferases maintain some genes in an active state. ATX1 regulates the timing of root development and is essential for stem cell niche maintenance and cell patterning during primary and lateral root development.

## Introduction

Gene expression changes to active or repressed states and the maintenance of these changes are essential for the orchestration of any developmental programme in living organisms. Trithorax (TrxG) and Polycomb group (PcG) proteins are important regulatory factors that contribute to the maintenance of gene expression states (Pien and Grossniklaus, 2007; Avramova, 2009; Alvarez-Venegas, 2010; Berr *et al.*, 2011; Schuettengruber *et al.*, 2011). Therefore, these proteins represent critical dynamic factors that act at the epigenetic level to define cell and tissue identities in both animal and plant species.

Numerous chromatin modification factors participate in various developmental processes and play essential roles in root development. Mutants in genes encoding two subunits of CHROMATIN ASSEMBLY FACTOR-1 (CAF-1), *fasciata1-1* (*fas 1-1*) and *fas 2-2*, are characterized by aberrant root apical meristem (RAM) organization. The quiescent centre (QC) of these mutants is either absent or difficult to identify (Kaya *et al.*, 2001). In addition, *FAS2* is involved in trichoblast/atrichoblast cell specification (Costa and Shaw, 2006) and *FAS1* in lateral root (LR) initiation (Manzano *et al.*, 2012). A histone deacetylase, HDA18, participates in root epidermal cell patterning (Xu *et al.*, 2005), and a histone acetyltransferase, GCN5 (GENERAL CONTROL NONDEREPRESSIBLE 5), and the GCN5-associated factor, ADA2b (ALTERATION/DEFICIENCY IN ACTIVATION 2B), act in the PLETHORA pathway and are essential for maintaining RAM activity (Kornet and Scheres, 2009). Furthermore, a SWI2/SNF2 chromatin-remodelling ATPase, BRAHMA, regulates primary root growth in an ABA-dependent manner (Han *et al.*, 2012). *PICKLE* (*PKL*), a chromatin-remodelling factor with the chromodomain/helicase/DNA-binding domain (CHD3/CHD4), is required for the transition from the embryonic stage to post-embryonic development (Ogas *et al.*, 1999), for repression of LR initiation through auxin-dependent negative regulation of pericycle cell activation (Fukaki *et al.*, 2006), and for RAM maintenance (Aichinger *et al.*, 2011). RAM activity is regulated by the antagonistic activity between a PcG protein, CURLY LEAF (CLF), and PKL (Aichinger *et al.*, 2011).

TrxG and PcG proteins are involved in maintaining the active and repressed states of genes with antagonistic functions (Köhler and Hennig, 2010). Most TrxG proteins exert their function as part of large multimeric protein complexes that have either histone-modifying or nucleosome-remodelling activities. Thus, TrxG proteins are involved in the formation of an open chromatin structure and facilitate transcription by being involved in chromatin remodelling (Breiling *et al.*, 2007). The best-studied member of this group in plants is *ARABIDOPSIS HOMOLOG of TRITHORAX1* (*ATX1/SDG27*), which is a member of the *SET DOMAIN GROUP* (*SDG*) family of genes and encodes a H3K4histone methyltransferase. *ATX1* participates in flower development by activating flower homeotic genes (Alvarez-Venegas *et al.*, 2003; Pien *et al.*, 2008). However, little is known about the role of TrxG genes in root development, and only recently was it shown that *SDG2*, which encodes another H3K4histone methyltransferase, is required for root growth and root stem cell niche maintenance (Yao *et al.*, 2013). Here it is shown that *ATX1* is essential for proper RAM organization, RAM activity, and, subsequently, for cell production. The morphogenesis of lateral root primordia (LRPs) of the *atx1-1* loss-of-function mutant was affected at both early and later developmental stages. These data suggest that this TrxG gene is required for cell proliferation-related processes, cell patterning, and morphogenesis of the root.

## Materials and methods

### Plant materials and growth conditions

*Arabidopsis thaliana* (L.) Heyhn wild type (Wt) and the *atx1-1* mutant were in the Wassilewskija (Ws) ecotype. The isolation and shoot phenotype of the *atx1-1* mutant have been described (Alvarez-Venegas *et al.*, 2003). Transgenic marker lines *pWOX5::GFP* (Sarkar *et al.*, 2007), *pSCR::GFP* (Heidstra *et al.*, 2004), *pDR5rev::GFP* (Friml *et al.*, 2003), and *Cyclin B1;1*_*DB*_ *::GUS* (Colón-Carmona *et al.*, 1999) have also been described. Seeds were sterilized for 10min in 20% commercial bleach and 0.08% Triton X-100, washed four times with sterile distilled water, and imbibed at 4 °C for 2 d. Plants were grown in soil (Metromix 200) or in Petri dishes oriented vertically and containing 0.2× Murashige and Skoog (MS) medium prepared from Linsmaier and Skoog medium (L477; Phyto Technology Laboratories, Lenexa, KS, USA), pH 5.7, and supplemented with vitamins (0.1mg l^–1^ pyridoxine, 0.1mg l^–1^ nicotinic acid, from Sigma-Aldrich, St Louis, MO, USA), 1% sucrose, and 0.8% agar (w/v, Bacto™ Agar; BD Difco, Sparks, MD, USA). All plants were grown at 21 °C, under a 16/8h light/dark photoperiod with a light intensity of 105 μmol photons m^–2^ s^–1^.

### Auxin treatments and RT–qPCR

For root growth assays, Wt and *atx1-1* seedlings were grown for 3 days post-germination (dpg) in vertically oriented Petri dishes containing 0.2× MS medium and then transferred to the same medium or media supplemented with 1 μM or 5 μM indole acetic acid (IAA), and grown for an additional 5 d. For transcript analysis, 7.5 dpg seedlings were treated with 1 μM naphthaleneacetic acid (NAA) for 12h and then RNA was extracted. IAA and NAA were purchased from Sigma-Aldrich. Total RNA was extracted from roots of Wt and *atx1-1* seedlings using TRIzol reagent (Invitrogen), according to the manufacturer’s instructions. Real-time quantitative reverse transcription–PCR (RT–qPCR) analysis was performed using an iQ5 Multicolor Real-time PCR Detection System (Bio-Rad, Hercules, CA, USA). Reactions were set up using a one-step RT-PCR Kit with SYBR Green (Bio-Rad), according to the manufacturer’s instructions; 100ng of RNA was used for each reaction, except for the negative control. The primers specific for AUX/*IAA14* (AT4G14550) were IAA14-Fw CCT CCT GCT AAA GCA CAA GTG and IAA14-Rv CTT CGC CGC TCT TCT GAT TAG C. Data were normalized to the expression of two reference genes, *UBQ10* (At4g05320) and *EF1α* (AT5G60390) (Czechowski *et al.*, 2005), and normalized expression levels were calculated according to Vandesompele *et al.* (2002). Two biological and six technical replicates were performed.

### Microscopy

Roots were cleared using an acidified methanol procedure (Malamy and Benfey, 1997) with modifications as described (Dubrovsky *et al.*, 2006), and whole-mount preparations were analysed under a Zeiss Axiovert 200M microscope (Zeiss, Oberkochen, Germany) equipped with differential interferential contrast (DIC; Nomarski) optics. For β-glucuronidase (GUS) staining, roots were pre-fixed in 0.3% formaldehyde for 20min at room temperature, washed in 100mM sodium phosphate buffer, pH 7.4, and stained as described (Hemerly *et al.*, 1993). Photographs were taken using a Photometrics CoolSNAPcf Color Camera (Valley International Corporation, Austin, TX, USA). The roots were fixed in 50% methanol and 10% acetic acid at 4 °C for 5h and pseudo-Schiff staining was performed as described (Truernit *et al.*, 2008). Then, samples were mounted in NaI-based clearing and mounting solution (Dubrovsky *et al.*, 2009). Live roots were stained with 1 μg mL^–1^ or 5 μg mL^–1^ propidium iodide dissolved in water. Confocal laser scanning microscopy (CLSM) was performed with a Zeiss LSM 510 Meta (Oberkochen, Germany) microscope using sequential scanning. For the red and green channels, the 543nm line of a He/Ne laser and the 488nm line of an Ar laser were used for excitation, respectively. In some instances (indicated in the figure legends), image contrast was improved using the Gaussian blur and Unshurp mask filters in ImageJ (http://rsb.info.nih.gov/ij).

### Growth analysis

The position of the root tip of vertically grown roots was marked every 24h and the Petri dishes were scanned and root growth increments measured using ImageJ. LR density, the density of LRPs, length of fully elongated cortical cells, LR initiation index, length of the root apical meristem (RAM), length of the proliferation domain (PD), and the length of the transition domain (TD) were determined on cleared roots as described (Dubrovsky *et al.*, 2009; Dubrovsky and Forde, 2012; Ivanov and Dubrovsky, 2013). Criteria for defining the PD and TD have been described (Ivanov and Dubrovsky, 2013; López-Bucio *et al.*, 2014). Briefly, the PD comprises cells that maintain proliferation activity and the TD comprises cells that have a very low probability of proliferating, but grow at the same rate as cells in the PD and have not yet started elongating rapidly. The domains were determined based on relative changes in the cell lengths observed on cleared root preparations. In the PD, the cell length commonly varies no more than 2-fold, and in the TD, cells are longer than the longest cells in the PD. In the elongation zone (EZ), the cell length starts to increase steadily and simultaneously in all tissues. The point at which this increase can be observed was defined as the distal (rootward) border between the TD and the EZ.

The position of the most distal (rootward) LRP and LR, as well the number of LRPs in the LR formation and branching zones was determined on cleared root preparations under a microscope equipped with DIC optics. Cortical cell length was determined for 10 cells per root on cleared preparations using an ocular micrometer. The root growth parameters and RAM activity, including cell cycle duration, were evaluated as described (Ivanov and Dubrovsky, 1997; López-Bucio *et al.*, 2014). Briefly, all the parameters were evaluated for each individual root. The cell production rate was calculated based on the rate of root growth and the length of fully elongated cells, and the cell cycle time was evaluated based on cell production and the number of cells in the PD, as described (Ivanov and Dubrovsky, 1997). The number of cells displaced from the cell proliferation domain (*N*_transit_) during a 24h period was estimated from the equation *N*_transit_=(24 ln2 *N*_PD_)*T* ^–1^, based on assumptions given in Ivanov and Dubrovsky (1997), where *N*_PD_ is the number of cells in the PD of the RAM and *T* is the average cell cycle time (h).

### Timing of LR formation

The timing of LR formation, defined as the period from LRP initiation to LR emergence, was estimated based on the rate of root growth (*V*, mm h^–1^) and the length of the LR formation zone (*L*_P_, mm). The latter comprises the portion of the root from the most rootward (i.e. closest to the root tip) LRP to the most rootward emerged LR (Dubrovsky and Forde, 2012). For each individual root, the following data were collected: (i) the root growth increments during the last 3 d; (ii) the distance from the root tip to the most rootward LRP (*L*_I_, mm); and (iii) *L*_P_. The latter two parameters were determined in cleared roots. The measurements were performed using ImageJ. Previously, it was observed that length *L*_I_ did not change significantly in seedlings of different ages (JGD, personal observation). Therefore, it was assumed that *L*_I_ was the same in a root at the moment of LRP initiation and when the primordium emerged as an LR. The time interval (days) between the two time points was calculated based on the growth increments. Root growth rate (*V*) during these days was evaluated and LR formation time (*T*_P_, *h*) was calculated as *T*_P_=*VL*_P_^–1^. When the growth rate was significantly different between the last and the first days of growth recorded, to decrease a calculation error, a fraction of the root portion *L*_Pf_ formed during a certain day of growth was determined and *T*_P_ was evaluated as the sum of separately calculated *T*_Pf_ intervals.

### LRP symmetry analysis

To estimate the LRP symmetry, only primordia in cleared roots positioned on a slide in the protoxylem plane (i.e. both protoxylem strands were clearly visible in the same focal plane) were analysed (Dubrovsky *et al.*, 2000). The length of the primordium base was measured and did not include pericycle cells at the primordium borders that did not divide periclinally. From the centre of the primordium base line, a perpendicular line was drawn that corresponded to the axis of the prospective LR. From the centre of this axis line, two radii were drawn parallel to the primordium base and measured. If these radii were of equal lengths, the LRP was considered to be symmetrical (asymmetry=0). When the radii were of unequal length, the longer (*r*_l_) and shorter (*r*_s_) radii were recognized. To estimate the percentage of asymmetry (*A*) for each primordium, the following equation was used: *A=r*_l_ – *r*_s_ (*r*_l_+*r*_s_)^–1^ 100, where *r*_l_ is the longer radius length and *r*_s_ is the shorter radius length. An average of *A* values was calculated for Wt and *atx1-1* LRPs. The statistical analysis was performed using SigmaPlot 12 (Systat Software, San Jose, CA, USA). The number of independent experiments in each case is indicated in the corresponding figure legend. The two-tailed Student’s *t*-test and Mann–Whitney rank sum test were used.

## Results

### *ATX1* is required for root growth and cell production in the RAM

**Fig. 1.**
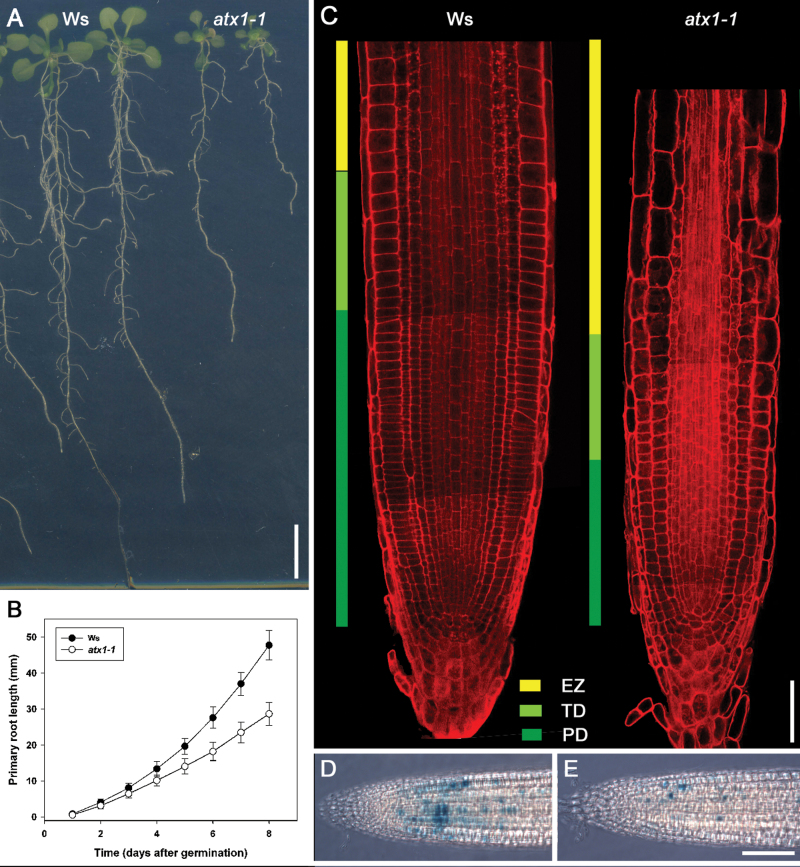
*ATX1* is required for primary root growth. (A) Wild-type (Ws) and *atx1-1* seedlings, at 15 days post-germination (dpg). (B) Primary root growth dynamics of Ws and *atx1-1* during the first 8 dpg. Values are means ±SD (*n*=27–32). Combined data of three independent experiments are shown. (C) Longitudinal zonation pattern in the primary roots of Ws and *atx1-1* seedlings at 8 dpg. Pseudo-Schiff-stained roots were analysed using confocal laser scanning microscopy. The proliferation domain (PD) and the transition domain (TD), which together form the root apical meristem (RAM), and the elongation zone (EZ) are colour-coded. (D and E) *CycB*_*DB*_ *1;1::GUS* expression in Ws (D) and *atx1-1* (E) seedlings at 8 dpg. Representative roots are shown (*n*=17–20 for each genotype). Scale bars=10mm (A), 50 μm (C), and 100 μm (D and E).

**Fig. 2.**
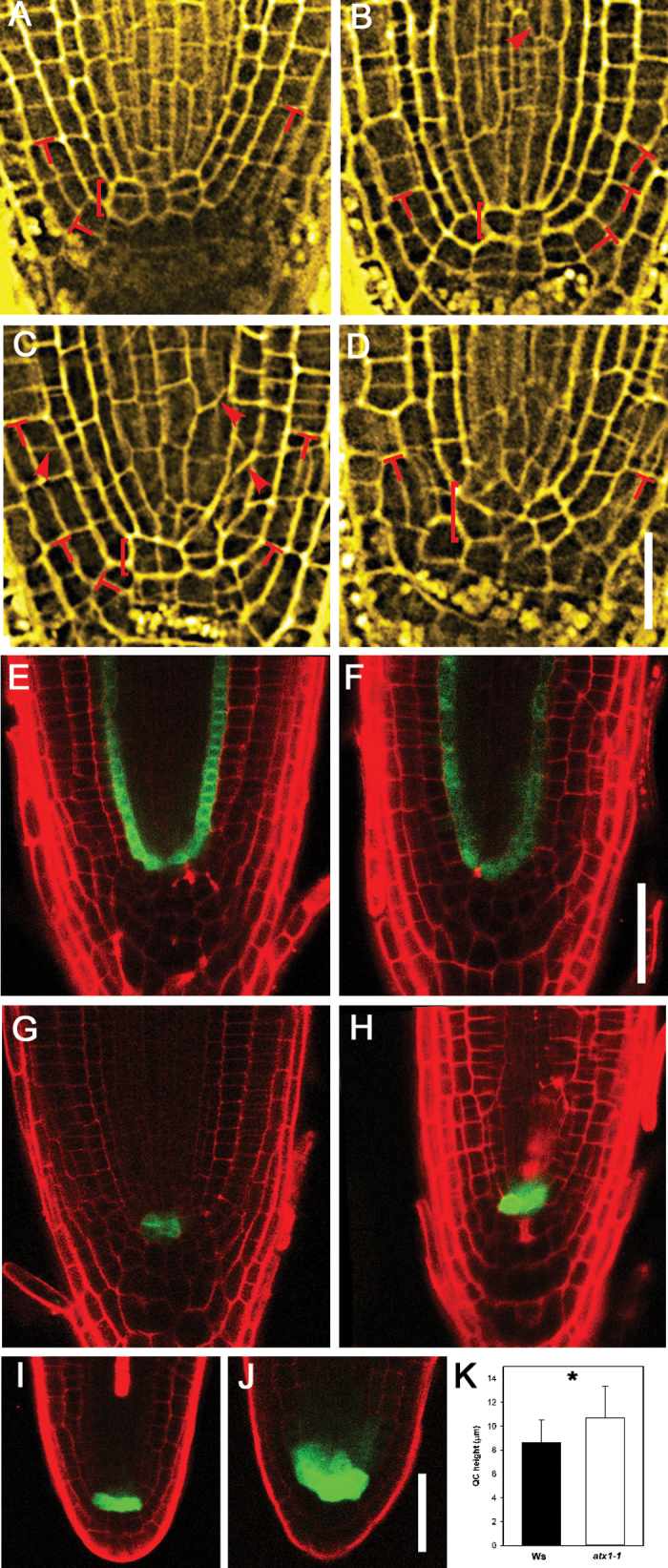
Cell patterning is altered in the primary root apical meristem (RAM) of *atx1-1*. (A–D) RAM organization in Ws (A) and *atx1-1* (B–D) plants. The organization shown in (A) was found in 20 out of 21 Wt plants analysed. In contrast, only one out of 22 *atx1-1* plants had a normal RAM organization (B), whereas the RAM was disorganized in the remaining 21 plants analysed (C and D). Square brackets indicate the quiescent centre (QC); T-divisions are indicated by red Ts; arrowheads indicate oblique divisions. (E, F) *pSCR::GFP* expression in the roots of Wt (E) and *atx1-1* (F) plants. (G–J) *pWOX5::GFP* expression in primary (G, H) and lateral (I, J) roots. (G, I) Wt plants. (H, J) *atx1-1*. (K) The QC height in Wt (Ws) and *atx1-1* plants. An asterisk indicates a statistically significant difference (*P*<0.05, *n*=16–19, Student’s *t*-test; error bars indicate the SD). In all cases, 8 dpg plants were analysed, and *n*=16–29 per genotype. (A–D) Pseudo-Schiff-stained, fixed roots were analysed using confocal laser scanning microscopy and shown after the application of Gaussian blur and Unshurp mask filters. (E–J) Red signal is from propidium iodide, which labels cell walls. Scale bars=20 μm (A–D), 50 μm (E–H) and (I) and (J).

A subset of histones present in the *atx1-1* loss-of-function mutant analysed in this study is known to be modified. Specifically, K4 methylation of histone H3 is significantly lower than in the Wt (Alvarez-Venegas and Avramova, 2005). The mutant exhibits abnormal flower development (Alvarez-Venegas *et al.*, 2003) and retarded root growth (Fig. 1A). The length of the primary root of the *atx1-1* mutant at 8 dpg was 60% of that of the Wt (Fig. 1B). Analysis of the longitudinal zonation pattern showed that the RAM was significantly shorter in the mutant (Fig. 1C). The cell proliferation domain (PD) and transition domain (TD) of the RAM (Ivanov and Dubrovsky, 2013) were clearly visible. The reduced RAM length was caused by a decrease in the length of the PD (Table 1). Confocal sections showed that the RAM cells of the *atx1-1* mutant were larger than those of the Wt (Figs 1C, 2), suggesting that cell division was delayed in the *atx1-1* RAM. To test the hypothesis that cell proliferation was affected in the mutant, the expression of a G_2_/M transition marker, *Cyclin B1;1*_*DB*_ *::GUS* (Colón-Carmona *et al.*, 1999), in the *atx1-1* background, was analysed (Fig. 1E). Indeed, far fewer RAM cells exhibited GUS activity in *atx1-1* than in the Wt, suggesting that cell proliferation activity was compromised in *atx1-1.*

**Table 1.** *Wild-type (Ws) and* atx1-1 *root growth and a comparative analysis of RAM activity in these two genotypes*All parameters were evaluated as indicated in the Materials and methods.

Genotype	Rate of root growth (μm h^–1^)	Elongated cell length (μm)	RAM length (μm)	PD length (μm)	PD no. of cells	TD length (μm)	Cell production rate (cell h^–1^)	Cell cycle duration (h)	*NC*_transit_ during 24 h
Ws	262±41	169±17	400±133	272±105	43±4	128±38	1.5±0.2	20.8±2.5	35±6
*atx1-1*	91±24	157±13	251±58	132±36	21±3	128±28	0.6±0.1	30.8±7.3	12±3
Statistics.	*	ND	*(NTF)	*	*	ND	*	*	*

Cell length of fully elongated cortical cells is indicated. Mean ±SD, *n* = 12.

*Statistical significance at *P*<0.001 (two-tailed Student’s *t*-test). When the normality test failed (NTF), a Mann–Whitney rank sum test was performed. ND indicates no significant difference (*P*>0.05).

To determine to what extent the cell proliferation activity of the RAM was affected in the *atx1-1* mutant, various parameters related to root growth and RAM activity were analysed (Table 1). Between 7 and 8 dpg, the growth rate of *atx1-1* roots was only 35% of that of the Wt. Fully elongated cell length was not affected in the mutant, indicating that decreased RAM activity was the main cause of the retarded root growth. Interestingly, while the length of the TD was the same in the Wt and *atx1-1*, the length of the *atx1-1* PD and the number of cells in this region were both 49% of those in the Wt. As a result, cell production by the RAM was 2.5-fold lower in the mutant than in the Wt (Table 1). The decreased activity of the RAM was also reflected in the increased cell cycle time. The cell cycle was 1.5 times longer in *atx1-1* than in the Wt.

RAM maintenance depends on a well-regulated balance between cell proliferation and the transition of cells to elongation (Ivanov, 1974, 1997; Barlow, 1976; Perilli *et al.*, 2012). Increased or decreased RAM size is thought to signify delayed or accelerated transition to elongation, respectively (e.g. Dello Ioio *et al.*, 2008). To determine if the decreased RAM length of the *atx1-1* mutant is related to an increased transition to elongation, the number of cells that start to elongate during the same time period in the mutant and Wt was estimated. It has been predicted that during one cell cycle, ln2 *N*_PD_ (i.e. ~70% of *N*_PD_) cells leave the PD of the RAM and become displaced to the TD and EZ of the RAM (Ivanov and Dubrovsky, 1997). Based on the estimated cell cycle duration, this approach was used to evaluate the number of cells that leave the RAM PD during a 24h period. Surprisingly, this analysis showed that the number of cells leaving the RAM PD is 2.9 times lower in the mutant than in the Wt (Table 1). Overall, this analysis suggests that *ATX1* modulates root growth by regulating the cell cycle time, cell production, and the transition from cell proliferation in the RAM to elongation. As RAM activity depends on the stem cell niche, it was thus important to establish whether the stem cell niche and RAM organization were altered in *atx1-*1.

### *ATX1* is required for the organization and cell patterning of the RAM and its stem cell niche

It was found that the organization of the QC and initial (stem) cells was irregular in *atx1-1* roots. Columella initial cells are recognized based on the absence of starch granules, which are present in differentiated columella cells. Columella initials in *atx1-1* formed one tier of cells similar to the Wt. However, in 21 out of 22 (95%) of the *atx1-1* roots examined, the QC was abnormal. Whereas QC cells are commonly transversely aligned in Wt roots (Fig. 2A; Dolan *et al.*, 1993), such an alignment was rarely found in the mutant roots, and the QC cells were frequently irregular in shape (Fig. 2B–D). From the second week after germination onwards, cells in the QC of Wt *Arabidopsis* seedlings of the Ws accession undergo periclinal divisions (Baum *et al.*, 2002). In agreement with this observation, periclinal divisions in the QC cells were observed in 12 of 16 (75%) Wt and 15 of 19 (80%) *atx1-1* seedlings at 8 dpg. As a result, the QC was composed of on average 1.8 cells in the longitudinal direction, and no statistical differences were found between the Wt and *atx1-1* plants (*n*=16–19, *P*=0.883, Mann–Whitney rank sum test). However, the QC height (the QC size in the longitudinal direction) was 19% greater in the mutant than in the Wt (Fig. 2K), indicating that the QC cells in *atx1-1* were more expanded than those in the Wt. Cell patterning above the QC was also strongly affected in the mutant, and aberrant oblique divisions in the provascular cylinder and ground tissue were not uncommon (arrowheads in Fig. 2B–D).

The root cap–protoderm (RCP) initial (stem) cell divides asymmetrically to give rise to cells with different cell type identities: protoderm (epidermis) and the lateral root cap (Kuras, 1978; Dolan *et al.*, 1993; Baum and Rost, 1996; Wenzel and Rost, 2001; Cruz-Ramírez *et al.*, 2012). Once the daughter cells have yielded the protoderm and the lateral root cap, the RCP stem cell divides again. When it undergoes a periclinal division (i.e. parallel to the nearest root surface), the division is recognized as a T-division (Kuras, 1978). This sequence of formative and proliferative division events is well coordinated, and T-divisions are therefore regularly distributed in the Wt (Baum and Rost, 1996; Wenzel and Rost, 2001).

This regularity is lost in *atx1-1*, and T-divisions are frequently observed in close proximity to each other (Fig. 2B, C). Cells in the PD mostly undergo anticlinal divisions (i.e. perpendicular to the nearest root surface). In all root tissues, including those of the provascular cylinder, many instances of atypical oblique divisions were found (Fig. 2C, arrowheads). Together, the irregularly shaped and enlarged QC, the atypical distribution of T-divisions, and the presence of oblique divisions in *atx1-1* suggest that a lack of coordination between cell division and cell growth results in abnormal cell patterning in *atx1-1* roots.

### *ATX1* restricts the expression domain of cells with quiescent centre identity

To establish whether these abnormalities affected cell type identities in the stem cell niche, various cell type marker lines were analysed. *SCARECROW* (*SCR*), a GRAS family transcription factor, is involved in RAM maintenance and radial patterning. It is expressed in the QC, cortex–endodermis initial (stem) cells, their daughters, and the endodermis (Di Laurenzio *et al.*, 1996; Nakajima *et al.*, 2001; Sabatini *et al.*, 2003). *WUSCHEL-RELATED HOMEOBOX 5* (*WOX5*) is specifically expressed in the QC (Sarkar *et al.*, 2007). To analyse possible changes in QC identity, *atx1-1* was crossed with the respective marker lines, and F_2_ seedlings carrying the marker in the *atx1-1* background were selected and propagated. The *pSCR::GFP* expression domain in *atx1-1* primary roots (*n*=17) was similar to that of Wt roots (Fig. 2F). *pWOX5::GFP* expression was observed in the QC of both Wt and *atx1* roots.

However, in 18 of 29 (62%) of the mutant plants analysed, the domain of *pWOX5* activity was expanded in the primary RAM compared with the Wt, as *pWOX5::GFP* was observed in the QC (as for the Wt) and also in provascular cells adjacent to the QC (Fig. 2G, H; Supplementary Fig. S1A–C available at *JXB* online). Moreover, in the RAM of first-order LRs, the *pWOX5::GFP* expression domain was expanded to an even greater extent (Fig. 2I, J) and was detected in 18 out of 22 (82%) LRs analysed. A QC46 QC marker was expressed in the QC in 13 out of 20 (65%) *atx1-1* primary roots, and the expression domain was expanded and detected in adjacent provascular cylinder cells (data not shown). Furthermore, it is known that RAM activity depends on established auxin gradients (Blilou *et al.*, 2005; Petersson *et al.*, 2009). To examine whether auxin response gradients in the RAM were affected in the mutant, the auxin-response marker *DR5rev::GFP* (Friml *et al.*, 2003) was introduced into the *atx1-1* background. Interestingly, *DR5rev::GFP* expression was unaltered in the *atx1-1* RAM (Supplementary Fig. S1D, E). Furthermore, primary root growth in *atx1-1* was inhibited by IAA to the same extent as in the Wt (Supplementary Fig. S2A, B). These results suggest that RAM abnormalities are auxin independent.

Collectively, the results indicated that *ATX1* is required for the maintenance of QC identity, RAM organization, and cell patterning. In particular, ATX1 contributes to the restriction of *WOX5* and *QC46* expression to the stem cell niche.

### *ATX1* is involved in LR emergence by controlling the timing and proliferation of LRP cells

**Fig. 3.**
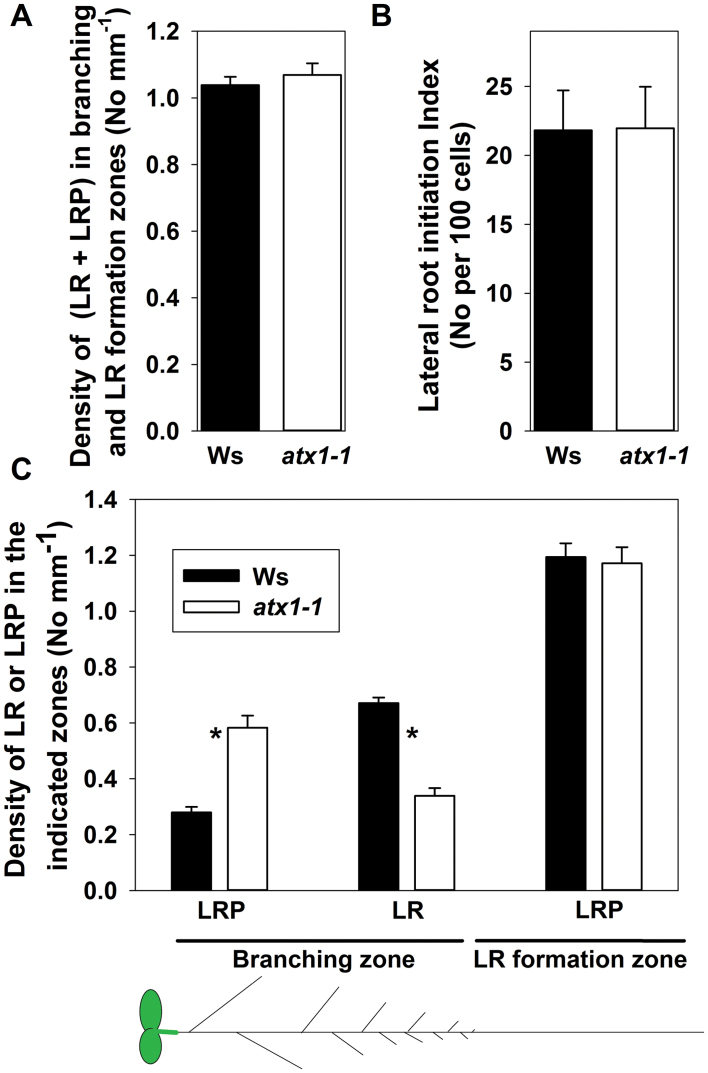
Quantitative analysis of lateral root (LR) formation in wild-type (Ws) and *atx1-1* plants. (A) Combined density of LR initiation events (including LRs and LRPs). (B) LR initiation index. The density and index were estimated within the branching and LR formation zones of the primary root. (C) LR and LRP density in the branching and LR formation zones. Mean ±SD, *n*=22, *P<0.001, Student’s *t*-test. The scheme at the bottom shows the branching zone and the LR formation zone of the seedling’s primary root.

As cell proliferation-related processes and cell patterning in the *atx1-1* mutant were affected during primary root development (Figs 1, 2; Table 1), it was of interest to analyse LR development in the *atx1-1* mutant. First, investigations were carried out to determine whether LRP initiation was affected. Despite retarded primary root growth, the density of all LR initiation events (including LRs and LRPs) was not affected in the mutant (Fig. 3A). Next, the LR initiation index was estimated; this is a parameter that evaluates the number of LR initiation events per root portion comprising 100 cortical cells of average length in a file (Dubrovsky *et al.*, 2009). As cell length was unaltered in *atx1-1* (Table 1), it was assumed that the LR initiation index would also be unchanged. Indeed, no differences was found in the index (Fig. 3B), indicating that LR initiation was not affected in the mutant. However, it was noticed that *atx1-1* had a less rooty phenotype (Fig. 1A). To characterize this phenotype quantitatively, the density of LRPs and LRs within the branching zone, which includes the root portion from the most distal (rootward) LR to the primary root base (Dubrovsky and Forde, 2012), was estimated. The LR density was significantly (2-fold) lower in the *atx1* mutant than in the Wt (Fig. 3C). As the density of all LR initiation events (including LRs and LRPs) in the mutant did not differ from those in the Wt (Fig. 3A), but the LR density in the branching zone decreased, it was expected that the density of LRPs within this zone would increase. Indeed, the LRP density was 2.1-fold greater in the branching zone of the mutant than in the corresponding region of the Wt (Fig. 3C). Thus, the LRP density in the LR formation zone (comprising the root portion from the most rootward LRP to the most rootward LR; Dubrovsky and Forde, 2012) confirmed that, quantitatively, LRP initiation was not affected in the mutant (Fig. 3C). Therefore, LR emergence but not initiation was affected in the mutant, and LRP development was slower in the *atx1-1* mutant than in the Wt.

**Fig. 4.**
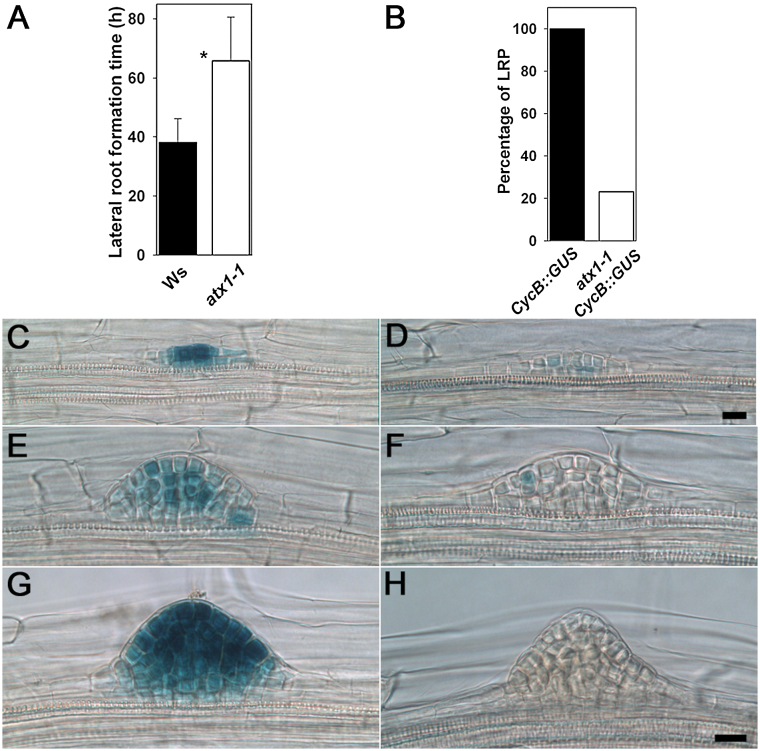
Timing of lateral root (LR) formation and cell proliferation in lateral root primordia (LRPs). (A) LR formation time from initiation to emergence in Wt (Ws) plants and the *atx1-1* mutant. An asterisk indicates statistical difference at *P*<0.001 (*n*=22–23); error bars indicate the SD. (B) Percentage of LRPs with detected expression of the *CycB1;1*_*DB*_ *::GUS* marker; *n*=83 LRPs in 20 seedlings and 32 LRPs in 14 seedlings of Wt and *atx1-1* background, respectively. (C, E, G) Expression of *CycB1;1*_*DB*_ *::GUS* in LRPs of Wt plants. (D, F, H) Expression of *CycB1;1*_*DB*_ *::GUS* in the LRPs of *atx1-1* plants. Scale bars=20 μm (C and D) and (E–H). (This figure is available in colour at *JXB* online.)

To explore whether the timing of LRP formation was affected in the *atx1-1* mutant, the period from LRP initiation to LR emergence was estimated using the approach proposed by Ivanov *et al.* (1998). This method permits time estimations from root growth rate (*V*, mm h^–1^) and the length of the LR formation zone (Ivanov *et al.*, 1998). This method is based on the supposition that the average distance from the root tip to the site of LRP initiation does not change with time (see details in the Materials and methods). The observations in *Arabidopsis* show that this is indeed the case (data not shown). LR formation time (i.e. the time from LR initiation to emergence) has not been evaluated for *Arabidopsis.* Here, it was demonstrated that this is a relatively rapid process that takes on average 38.1h in the Wt. In *atx1-1*, however, this time was 1.7-fold greater (Fig. 4A). Slower LR formation in the mutant compared with the Wt may explain the decreased root branching phenotype.

Cell production was affected in the *atx1-1* primary RAM (Table 1), and LR formation in the mutant was also slower than in the Wt. Therefore, it was hypothesized that cell proliferation may also be compromised during LRP formation. To test this possibility, the expression of the *CycB1;1*_*DB*_ *::GUS* G_2_/M transition marker (Colón-Carmona *et al.*, 1999) was analysed in the *atx1-1* background. Only 23% of LRP within the *atx1-1* LR formation zone showed detectable GUS staining (Fig. 4B). Even in primordia where GUS-positive cells were found, the number of such cells was much lower than in the Wt (Fig. 4C–H). Overall, these data suggested that *ATX1* regulates the timing of LRP development, apparently through its involvement in cell proliferation. As the data indicated that *ATX1* is important for cell patterning in the primary root meristem, and cell proliferation and patterning are frequently coupled, how cell patterning is affected during LRP morphogenesis was next studied.

### *ATX1* is required for LRP morphogenesis independently of auxin response

**Fig. 5.**
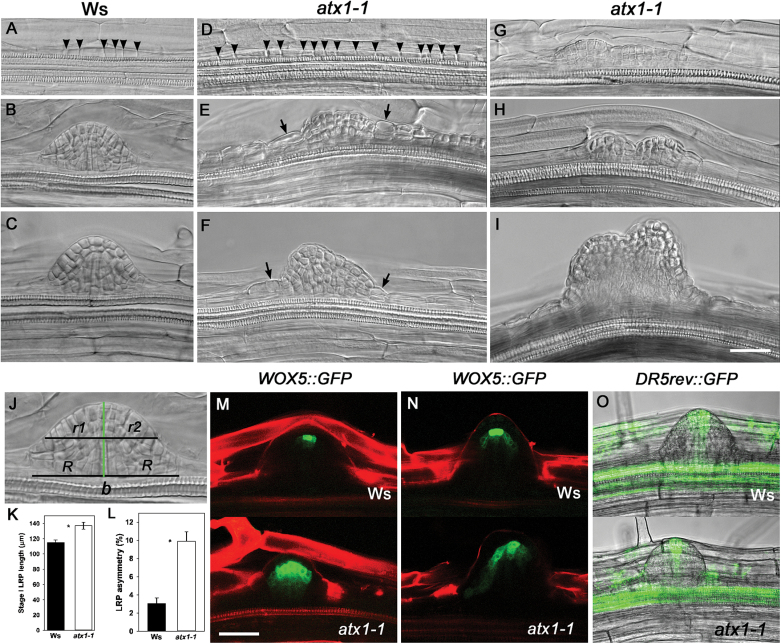
*ATX1* is required for lateral root primordium (LRP) morphogenesis. Nomarski images of LRP development in the Wt (Ws, A–C) and *atx1-1* (D–I). (G–I) Fused primordia in *atx1-1*. Arrowheads indicate anticlinal division in stage I primordia; arrows indicate enlarged cells at the lateral borders of the primordium. (J) A scheme showing how primordium asymmetry was evaluated. A primordium base (*b*) length was measured; from the centre of the primordium base, a perpendicular line corresponding to the longitudinal axis of a developing primordium was drawn (green line). From the centre of this longitudinal axis, two radii, *r*1 and *r*2, were drawn and measured. When the radii had different lengths, the longer radius was considered as *r*_l_ and the shorter *r*_s_. The percentage of asymmetry for each primordium was calculated as *r*_l_ –*r*_s_ (*r*_l_ +*r*_s_)^–1^ 100; (K) stage I LRP length in Ws and *atx1-1* plants. *P<0.003, Student’s *t*-test; error bars indicate the SE, *n*=91 LRPs in Ws and 79 LRPs in *atx1-1*. (L) LRP asymmetry percentage. *P<0.001, Student’s *t*-test; mean ±SE, *n*=25 LRPs in Ws and 39 LRPs in *atx1-1*. (M and N) *pWOX5::GFP* expression in Ws and *atx1-1* before (M) and after (N) LR emergence. (O) *DR5rev::GFP* expression in Ws and *atx1-1* plants at 8 dpg. Scale bar=10 μm (A–I), 50 μm (M–O).

Even though the incidence of LRP initiation was unaffected in the primary root of the mutant (Fig. 3), a detailed analysis showed that LRP initiation and development were affected in terms of morphogenesis. The first anticlinal divisions in the pericycle usually result in the formation of a core of a few cells that form an LR primordium (five cells in Fig. 5A). In *atx1-1*, such a core was frequently missing and stage I LRPs were much longer than in the Wt and included more cells (e.g. five cells in the Wt in Fig. 5A and 15 cells in *atx1-1* in Fig. 5D, see also Fig. 5K; LR developmental stages were defined as in Malamy and Benfey, 1997). As not all activated pericycle cells contribute equally to the formation of the dome of the LRP, the primordium shape of *atx1-1* was affected. Cells located near the longitudinal boundaries of the LRP were delayed in proliferation and were abnormally large (Fig. 5E, F). Developing LRPs were asymmetric (Fig. 5E, F). These abnormalities were found in 71% of all LRPs analysed (*n*=324 LRPs in 22 seedlings). To estimate the LRP asymmetry, *r*1 and *r*2 radii in developing stage V–VII LRPs of mid height were measured as shown (Fig. 5J), and the asymmetry value (*A*) was calculated as described in the Materials and methods. Asymmetry was much greater in the *atx1-1* mutant than in the Wt (Fig. 5L). In rare cases (two seedlings out of 22), not only a central group of cells, but almost all cells derived from the activated pericycle in stage I LRPs participated in LR body formation and two domes (apices) were formed that appeared to be fused (Fig. 5G–I). Similar abnormalities were never detected in the Wt. This analysis showed that patterning was affected in both the early and late stages of LRP formation.

As abnormal LRP morphogenesis was common in *atx1-1*, experiments were conducted to examine how this was related to QC establishment during LRP formation. It was found that in Wt seedlings, at LRP stages V–VII the expression of the QC marker *pWOX5::GFP* was restricted to QC cells, while a greater number of cells expressed the marker in *atx1-1* (Fig. 5M). Similarly, a greater domain of *pWOX5::GFP-*expressing cells was found in recently emerged LRs of the mutant (Fig. 5N). These observations suggest that QC establishment was affected in LRPs of the *atx1-1* mutant. Morphogenetic abnormalities in LRP development could be related to abnormal stem cell activities during the establishment of a new RAM. As auxin gradients are important for the RAM activity and the QC cell identity (Sabatini *et al.*, 1999; Aida *et al.*, 2004; Blilou *et al.*, 2005), the expression of the auxin reporter, *DR5rev::GFP*, was also analysed. No apparent change in GFP (green fluorescent protein) expression was found in stage V–VII LRPs or in recently emerged LRs (Fig. 5O). *AUX/IAA14* is a key auxin response gene involved in LR formation (Fukaki *et al.*, 2005). As expected, the level of *AUX/IAA14* expression increased upon auxin treatment; however, the expression of *AUX/IAA14* did not differ in the roots of Wt and *atx1-1* plants, either with or without auxin treatment (Supplementary Fig. S2E at *JXB* online). Therefore, this analysis showed that ATX1 controls cell patterning during LR development and that the developmental abnormalities of the *atx1-1* mutant are apparently unrelated to auxin response gradients.

## Discussion

Here the role of the most extensively studied *Arabidopsis* TrxG gene, *ATX1* (Avramova, 2009), was explored, and it was shown that, in addition to its known role in flower development, *ATX1* is an important player in root development. The data indicate that *ATX1* is required for primary root growth through its role in maintaining RAM activity and the transition to elongation, but not in cell elongation itself (Table 1). The RAM activity in the mutant was compromised in part by its increased cell cycle time. The fact that neither the transition domain of the RAM nor the fully elongated cells were affected in *atx1-1* underlines the importance of *ATX1* for cell proliferation-related processes and cell production. Interestingly, *SDG2* is also required for root growth, and the differences between the *sdg2* mutant and the Col Wt were similar to those between *atx1-1* and the Ws Wt (Guo *et al.*, 2010; Yao *et al.*, 2013). It has been shown that SDG2 is a major histone methyltransferase that contributes to the genome-wide H3K4me3 modifications in *Arabidopsis* (Guo *et al.*, 2010). Therefore, this and other histone methyltransferases are expected to be functional in *atx1-1.* In spite of this, the cell proliferation and cell patterning defects were found in *atx1-1* roots. This suggests that different methyltransferases are involved in regulating different aspects of developmental processes and implies non-redundant requirements of *ATX1* for root development.

It has been proposed that cell proliferation in the RAM and the transition from proliferation to elongation are regulated in the root independently (Ivanov, 1974, 1981, 1997). Decreased RAM length does not necessarily indicate accelerated cell differentiation as is sometimes considered (e.g. Dello Ioio *et al.*, 2008). Evaluation of how many cells during the same time period are displaced from the RAM to the TD and the EZ in the Wt and *atx1-1* would provide an estimate of whether the transition to elongation is affected in the mutant. This parameter was evaluated and a significant decrease in *atx1-1* was found compared with the Wt (Table 1). These results suggest that ATX1 modulates both cell proliferation and the transition to elongation. In studies of floral timing, it has been proposed that co-localization of the binding sites of an activating transcription factor and a Polycomb response element, which result in competition between PcG proteins and a transcription factor, may represent a general mechanism for timing regulation of cell division-dependent processes (Sun *et al.*, 2014). The present findings that *ATX1* is required to maintain cell cycle timing in the RAM and is involved in regulating the transition to elongation suggest that TrxG members may be involved in controlling the timing-related processes of RAM development.

RAM activity depends on stem cell activity. Descendants of stem cells either differentiate or maintain a proliferation-competent state. This decision is mediated by the balanced activity of PcG and TrxG proteins (Köhler and Hennig, 2010). Abnormal RAM organization in the *atx1-1* strongly suggests that *ATX1* is required for stem cell activity in the root. The facts that the QC was larger in *atx1-1* and that *pWOX5::GFP* and QC46 expression domains were expanded compared with the Wt indicate that the QC identity was compromised in the *atx1-1* mutant. This in turn may explain the abnormalities observed in initial (stem) cells, as their activity is dependent on QC cells (Van Den Berg *et al.*, 1995). There is a distinct difference between stem cell organization in *sdg2* and *atx1-1* mutants: in contrast to *atx1-1* (Fig. 2), columella initial (stem) cells are differentiated in *sdg2-3* roots (Yao *et al.*, 2013). However, the QC cells in *sdg2-3* do not lose their identity, similar to those of *atx1-1* (Yao *et al.*, 2013), and both mutants maintain a functional RAM, although with different degrees of abnormalities. In Wt roots, the auxin concentration is maximal in the QC (Petersson *et al.*, 2009) and the distal auxin gradients are involved in maintaining RAM activity (Blilou *et al.*, 2005). In spite of a number of abnormalities in the *atx1-1* RAM, the auxin response was unaltered (Supplementary Fig. S1 at *JXB* online), in contrast to *sdg2-3* (Yao *et al.*, 2013). This observation confirms that these TrxG genes perform at least some non-redundant functions.

Overall, the analysis of the role of *ATX1* in the RAM suggests that it participates in cell proliferation and cell patterning processes. This conclusion was confirmed in analyses of developing *atx1-1* LRs. Similar to primary root development, LRP development was slow in the *atx1-1* mutant. Considering that LR formation largely depends on cell proliferation and that the cell cycle time during LRP morphogenesis is very short (Dubrovsky *et al.*, 2001), an increase in the period from LR initiation to LR emergence in *atx1-1* indicates that cell proliferation in the LRP was also affected. Despite the role of *ATX1* in cell proliferation demonstrated here, the rate of LR initiation was unaffected in the *atx1-1* mutant, signifying that ATX1 has differential roles in distinct developmental processes. Nevertheless, LR initiation was affected in terms of early primordium morphogenesis, as abnormally wide primordia were formed (Fig. 5). The chromatin-remodelling factor PICKLE (PKL) is required for primary root growth, as it maintains stem cell activity and the size of the RAM. It is also required to maintain the active state of genes involved in RAM activity, such as *PLT1*, *PLT2*, *WOX5*, and *AGL42* (Aichinger *et al.*, 2011). Nevertheless, in the *pkl* mutant, although *pWOX5::GFP* expression is reduced, it is restricted to the QC (Aichinger *et al.*, 2011), whereas in the *atx1-1* mutant, the *pWOX5::GFP* expression domain is expanded during both primary root and LR development. The increased *pWOX5::GFP* expression domain in the developing LRP of *atx1-1* plants suggested that this mutant had abnormal or delayed QC establishment that could be responsible for the abnormal primordium morphogenesis.

To conclude, it is suggested that the morphological defects found in the *atx1-1* roots are related to the observed defects in cell proliferation. For example, unusually large cells at the LRP boundaries (Fig. 5) could be the result of the increased cell cycle time and continued cell growth. If interphase cell growth is not constant for all proliferating cells, it may result in LRP asymmetry. Similarly, grouped T-divisions in the RAM (Fig. 2) may also be a consequence of increased cell cycle duration and loss of coordination between cell division and growth. As mentioned above, the QC function in the RAM and its establishment during LR formation are compromised in *atx1-1*. LRP morphogenesis is affected by a number of factors, many of which are auxin related (reviewed in Szymanowska-Pułka, 2013). Importantly, the abnormalities identified in the LRP and RAM patterning of *atx1-1* were apparently unrelated to the auxin response. It remains to be determined which genes regulated by ATX1 are important for root growth and development.

## Supplementary data

Supplementary data are available at *JXB* online.

Figure S1. Expansion of the *pWOX5::GFP* expression domain and the auxin response in the primary root apical meristem of the *atx1-1 mutant.*

Figure S2. Effect of auxin on root development in Wt (Ws) and *atx1-1* plants.

Supplementary Data

## Abbreviations:

ATX1: *ARABIDOPSIS HOMOLOG of TRITHORAX1*
dpg: days post-germination
LR: lateral root
LRP: lateral root primordium
PcG: Polycomb group
PD: proliferation domain
QC: quiescent centre
RAM: root apical meristem
RCP: root cap–protoderm initial (stem) cell
TD: transition domain
TrxG: Trithorax group
Wt: wild type.

## References

[CIT0001] Aichinger E Villar CBR Di Mambro R Sabatini S Köhler C 2011 The CHD3 chromatin remodeler PICKLE and Polycomb Group proteins antagonistically regulate meristem activity in the *Arabidopsis* root. The Plant Cell 23, 1047–1060. 21441433 10.1105/tpc.111.083352 PMC3082253

[CIT0002] Aida M Beis D Heidstra R Willemsen V Blilou I Galinha CR Nussaume L Noh Y-S Amasino R Scheres B 2004 The *PLETHORA* genes mediate patterning of the *Arabidopsis* root stem cell niche. Cell 119, 109–120. 15454085 10.1016/j.cell.2004.09.018

[CIT0003] Alvarez-Venegas R 2010 Regulation by Polycomb and Trithorax Group proteins in Arabidopsis. The Arabidopsis Book 8, e0128. 22303254 10.1199/tab.0128 PMC3244960

[CIT0004] Alvarez-Venegas R Avramova Z 2003 Methylation patterns of histone H3 Lys 4, Lys 9 and Lys 27 in transcriptionally active and inactive *Arabidopsis* genes and in *atx1* mutants. Nucleic Acids Research 33, 5199–5207. 16157865 10.1093/nar/gki830 PMC1214549

[CIT0005] Alvarez-Venegas R Pien S Sadder M Witmer X Grossniklaus U Avramova Z 2003 ATX-1, an *Arabidopsis* homolog of Trithorax, activates flower homeotic genes. Current Biology 13, 627–637. 12699618 10.1016/s0960-9822(03)00243-4

[CIT0006] Avramova Z 2009 Evolution and pleiotropy of TRITHORAX function in *Arabidopsis* . International Journal of Developmental Biology 53, 371–381. 19412892 10.1387/ijdb.082664za

[CIT0007] Barlow PW 1976 Towards an understanding of the behaviour of root meristems. Journal of Theoretical Biology 57, 433–451. 957672 10.1016/0022-5193(76)90014-x

[CIT0008] Baum SF Dubrovsky JG Rost TL 2002 Apical organization and maturation of the cortex and vascular cylinder in *Arabidopsis thaliana* (Brassicaceae) roots. American Journal of Botany 89, 908–920. 21665690 10.3732/ajb.89.6.908

[CIT0009] Baum SF Rost TL 1996 Root apical organization in *Arabidopsis thaliana*. 1. Root cap and protoderm. Protoplasma 192, 178–188.

[CIT0010] Berr A Shafiq S Shen W-H 2011 Histone modifications in transcriptional activation during plant development. Biochimica et Biophysica Acta 1809, 567–576. 21777708 10.1016/j.bbagrm.2011.07.001

[CIT0011] Blilou I Xu J Wildwater M Willemsen V Paponov I Friml J Heidstra R Aida M Palme K Scheres B 2005 The PIN auxin efflux facilitator network controls growth and patterning in *Arabidopsis* roots. Nature 433, 39–44. 15635403 10.1038/nature03184

[CIT0012] Breiling A Sessa L Orlando V 2007 Biology of Polycomb and Trithorax group proteins. International Review of Cytology 258, 83–136. 17338920 10.1016/S0074-7696(07)58002-2

[CIT0013] Colón-Carmona A You R Haimovitch-Gal T Doerner P 1999 Spatio-temporal analysis of mitotic activity with a labile cyclin–GUS fusion protein. The Plant Journal 20, 503–508. 10607302 10.1046/j.1365-313x.1999.00620.x

[CIT0014] Costa S Shaw P 2006 Chromatin organization and cell fate switch respond to positional information in Arabidopsis. Nature 439, 493–496. 16362059 10.1038/nature04269

[CIT0015] Cruz-Ramírez A Díaz-Triviño S Blilou I 2012 A bistable circuit involving SCARECROW–RETINOBLASTOMA integrates cues to inform asymmetric stem cell division. Cell 150, 1002–1015. 22921914 10.1016/j.cell.2012.07.017 PMC3500399

[CIT0016] Czechowski T Stitt M Altmann T Udvardi MK Scheible WR 2005 Genome-wide identification and testing of superior reference genes for transcript normalization in *Arabidopsis*. Plant Physiology 139, 5–17. 16166256 10.1104/pp.105.063743 PMC1203353

[CIT0017] Dello Ioio RD Nakamura K Moubayidin L Perilli S Taniguchi M Morita MT Aoyama T Costantino P Sabatini S 2008 A genetic framework for the control of cell division and differentiation in the root meristem. Science 322, 1380–1384. 19039136 10.1126/science.1164147

[CIT0018] Di Laurenzio L Wysocka-Diller J Malamy JE Pysh L Helariutta Y Freshour G Hahn MG Feldmann KA Benfey PN 1996 The SCARECROW gene regulates an asymmetric cell division that is essential for generating the radial organization of the Arabidopsis root. Cell 86, 423–433. 8756724 10.1016/s0092-8674(00)80115-4

[CIT0019] Dolan L Janmaat K Willemsen V Linstead P Poethig S Roberts K 1993 Cellular organisation of the Arabidopsis thaliana root. Development 119, 71–84. 8275865 10.1242/dev.119.1.71

[CIT0020] Dubrovsky JG Doerner PW Colón-Carmona A Rost TL 2000 Pericycle cell proliferation and lateral root initiation in Arabidopsis. Plant Physiology 124, 1648–1657. 11115882 10.1104/pp.124.4.1648 PMC59863

[CIT0021] Dubrovsky JG Forde BG 2012 Quantitative analysis of lateral root development: pitfalls and how to avoid them. The Plant Cell 24, 4–14. 22227889 10.1105/tpc.111.089698 PMC3289558

[CIT0022] Dubrovsky JG Gambetta GA Hernández-Barrera A Shishkova S González I 2006 Lateral root initiation in Arabidopsis: developmental window, spatial patterning, density and predictability. Annals of Botany 97, 903–915. 16390845 10.1093/aob/mcj604 PMC2803408

[CIT0023] Dubrovsky JG Rost TL Colón-Carmona A Doerner PW 2001 Early primordium morphogenesis during lateral root initiation in *Arabidopsis thaliana* . Planta 214, 30–36. 11762168 10.1007/s004250100598

[CIT0024] Dubrovsky JG Soukup A Napsucialy-Mendivil S Jeknic Z Ivanchenko MG 2009 The lateral root initiation index: an integrative measure of primordium formation. Annals of Botany 103, 807–817. 19151042 10.1093/aob/mcn267 PMC2707874

[CIT0025] Friml F Vieten A Sauer M Weijers D Schwarz H Hamann T Offringa R Jürgens G 2003 Efflux-dependent auxin gradients establish the apical–basal axis of *Arabidopsis* . Nature 426, 147–153. 14614497 10.1038/nature02085

[CIT0026] Fukaki H Nakao Y Okushima Y Theologis A Tasaka M 2005 Tissue-specific expression of stabilized SOLITARYROOT/IAA14 alters lateral root development in Arabidopsis. The Plant Journal 44, 382–395. 16236149 10.1111/j.1365-313X.2005.02537.x

[CIT0027] Fukaki H Taniguchi N Tasaka M 2006 PICKLE is required for SOLITARY-ROOT/IAA14-mediated repression of ARF7 and ARF19 activity during Arabidopsis lateral root initiation. The Plant Journal 48, 380–389. 17010112 10.1111/j.1365-313X.2006.02882.x

[CIT0028] Guo L Yu Y Law JA Zhang X 2010 SET DOMAIN GROUP2 is the major histone H3 lysine 4 trimethyltransferase in *Arabidopsis* . Proceedings of the National Academy of Sciences, USA 107, 18557–18562. 10.1073/pnas.1010478107 PMC2972934 20937886

[CIT0029] Han S-K Sang Y Rodrigues A F B Wu M-F Rodriguez PL Wagner D 2012 The SWI2/SNF2 chromatin remodeling ATPase BRAHMA represses abscisic acid responses in the absence of the stress stimulus in *Arabidopsis* . The Plant Cell 24, 4892–4906. 23209114 10.1105/tpc.112.105114 PMC3556964

[CIT0030] Heidstra R Welch D Scheres B 2004 Mosaic analyses using marked activation and deletion clones dissect *Arabidopsis* SCARECROW action in asymmetric cell division. Genes and Development 18, 1964–1969. 15314023 10.1101/gad.305504 PMC514176

[CIT0031] Hemerly AS Ferreira P de Almeida Engler J Van Montagu M Engler G Inzé D 1993 *cdc2a* expression in Arabidopsis is linked with competence for cell division. The Plant Cell 5, 1711–1723. 8305869 10.1105/tpc.5.12.1711 PMC160398

[CIT0032] Ivanov V 1974 Cellular bases of plant growth. Moscow: Nauka.

[CIT0033] Ivanov V 1981 Cellular basis of root growth. Soviet Scientific Reviews. Section D. Biology Reviews.

[CIT0034] Ivanov VB 1997 Relationship between cell proliferation and transition to elongation in plant roots. International Journal of Developmental Biology 41 907–915. 9449467

[CIT0035] Ivanov VB Bystrova EI Dubrovsky JG Ploshinskaya ME 1998 Duration of lateral root formation in maize seedlings as affected by diverse factors. In: Box JE Jr, ed. Root demographics and their efficiencies in sustainable agriculture, grasslands and forest ecosystems. Dordrecht, The Netherlands: Kluwer Academic Publishers, 777–787.

[CIT0036] Ivanov VB Dubrovsky JG 1997 Estimation of the cell-cycle duration in the root meristem: a model of linkage between cell-cycle duration, rate of cell production, and rate of root growth. International Journal of Plant Sciences 158, 757–763.

[CIT0037] Ivanov VB Dubrovsky JG 2013 Longitudinal zonation pattern in plant roots: conflicts and solutions. Trends in Plant Science 18, 237–243. 23123304 10.1016/j.tplants.2012.10.002

[CIT0038] Kaya H Shibahara KI Taoka KI Iwabuchi M Stillman B Araki T 2001 FASCIATA genes for chromatin assembly factor-1 in *Arabidopsis* maintain the cellular organization of apical meristems. Cell 104, 131–142. 11163246 10.1016/s0092-8674(01)00197-0

[CIT0039] Köhler C Hennig L 2010 Regulation of cell identity by plant Polycomb and trithorax group proteins. Current Opinion in Genetics and Development 20, 541–547. 20684877 10.1016/j.gde.2010.04.015

[CIT0040] Kornet N Scheres B 2009 Members of the GCN5 histone acetyltransferase complex regulate PLETHORA-mediated root stem cell niche maintenance and transit amplifying cell proliferation in *Arabidopsis* . The Plant Cell 21, 1070–1079. 19376933 10.1105/tpc.108.065300 PMC2685635

[CIT0041] Kuras M 1978 Activation of embryo during rape (Brassica napus L.) seed germination I. Structure of embryo and germination of root apical meristem. Acta Societatis Botanicorum Poloniae 47, 65–82.

[CIT0042] López-Bucio JS Dubrovsky JG Raya-González J Ugartechea-Chirino Y López-Bucio J de Luna-Valdez LA Ramos-Vega M León P Guevara-García AA 2014 *Arabidopsis thaliana* mitogen-activated protein kinase 6 is involved in seed formation and modulation of primary and lateral root development. Journal of Experimental Botany 65, 169–183. 24218326 10.1093/jxb/ert368 PMC3883294

[CIT0043] Malamy JE Benfey PN 1997 Organization and cell differentiation in lateral roots of *Arabidopsis thaliana* . Development 124, 33–44. 9006065 10.1242/dev.124.1.33

[CIT0044] Manzano C Ramirez-Parra E Casimiro I Otero S Desvoyes B De Rybel B Beeckman T Casero P Gutierrez C del Pozo JC 2012 Auxin and epigenetic regulation of SKP2B, an F-Box that represses lateral root formation. Plant Physiology 160, 749–762. 22837358 10.1104/pp.112.198341 PMC3461553

[CIT0045] Nakajima K Sean G Nawy T Benfey PN 2001 Intercellular movement of the putative transcription factor SHR in root patterning. Nature 413, 307–311. 11565032 10.1038/35095061

[CIT0046] Ogas J Kaufmann S Henderson J Somerville C 1999 PICKLE is a CHD3 chromatin-remodeling factor that regulates the transition from embryonic to vegetative development in Arabidopsis. Proceedings of the National Academy of Sciences, USA 96, 13839–13844. 10.1073/pnas.96.24.13839 PMC24151 10570159

[CIT0047] Perilli S Di Mambro R Sabatini S 2012 Growth and development of the root apical meristem. Current Opinion in Plant Biology 15, 17–23. 22079783 10.1016/j.pbi.2011.10.006

[CIT0048] Petersson SV Johansson AI Kowalczyk M Makoveychuk A Wang JY Moritz T Grebe M Benfey PN Sandberg G Ljung K 2009 An auxin gradient and maximum in the *Arabidopsis* root apex shown by high-resolution cell-specific analysis of IAA distribution and synthesis. The Plant Cell 21, 1659–1668. 19491238 10.1105/tpc.109.066480 PMC2714926

[CIT0049] Pien S Fleury D Mylne JS Crevillen P Inze D Avramova Z Dean C Grossniklaus U 2008 ARABIDOPSIS TRITHORAX1 dynamically regulates *FLOWERING LOCUS C* activation via histone 3 lysine 4 trimethylation. The Plant Cell 20, 580–588. 18375656 10.1105/tpc.108.058172 PMC2329943

[CIT0050] Pien S Grossniklaus U 2007 Polycomb group and trithorax group proteins in Arabidopsis. Biochimica et Biophysica Acta 1769, 375–382. 17363079 10.1016/j.bbaexp.2007.01.010

[CIT0051] Sabatini S Beis D Wolkenfelt H 1999 An auxin-dependent distal organizer of pattern and polarity in the *Arabidopsis* root. Cell 99, 463–472. 10589675 10.1016/s0092-8674(00)81535-4

[CIT0052] Sabatini S Heidstra R Wildwater M Scheres B 2003 SCARECROW is involved in positioning the stem cell niche in the Arabidopsis root meristem. Genes and Development 17, 354–358. 12569126 10.1101/gad.252503 PMC195985

[CIT0053] Sarkar AK Luijten M Miyashima S Lenhard M Hashimoto T Nakajima K Scheres B Heidstra R Laux T 2007 Conserved factors regulate signalling in *Arabidopsis thaliana* shoot and root stem cell organizers. Nature 446, 811–814. 17429400 10.1038/nature05703

[CIT0054] Schuettengruber B Martinez AM Iovino N Cavalli G 2011 Trithorax group proteins: switching genes on and keeping them active. Nature Reviews Molecular Cell Biology 12, 799–814. 10.1038/nrm3230 22108599

[CIT0055] Sun B Looi L-S Guo S He Z Gan E-S Huang J Xu Y Wee W-Y Ito T 2014 Timing mechanism dependent on cell division is invoked by Polycomb eviction in plant stem cells. Science 343, 1248559. 24482483 10.1126/science.1248559

[CIT0056] Szymanowska-Pułka J 2013 Form matters: morphological aspects of lateral root development. Annals of Botany 112, 1643–1654. 24190952 10.1093/aob/mct231 PMC3838556

[CIT0057] Truernit E Bauby H Dubreucq B Grandjean O Runions J Barthelemy J Palauqui JC 2008 High-resolution whole-mount imaging of three-dimensional tissue organization and gene expression enables the study of phloem development and structure in *Arabidopsis* . The Plant Cell 20, 1494–1503. 18523061 10.1105/tpc.107.056069 PMC2483377

[CIT0058] Van Den Berg C Willemsen V Hage W Weisbeek P Scheres B 1995 Cell fate in the *Arabidopsis* root meristem determined by directional signalling. Nature 378, 62–65. 7477287 10.1038/378062a0

[CIT0059] Vandesompele J De Preter K Pattyn F Poppe B Van Roy N De Paepe A Speleman F 2002 Accurate normalization of real-time quantitative RT-PCR data by geometric averaging of multiple internal control genes. Genome Biology 3, research0034.0031. 10.1186/gb-2002-3-7-research0034 PMC126239 12184808

[CIT0060] Wenzel CL Rost TL 2001 Cell division patterns of the protoderm and root cap in the ‘closed’ root apical meristem of *Arabidopsis thaliana.* Protoplasma 218, 203–213. 11770436 10.1007/BF01306609

[CIT0061] Xu C-R Liu C Wang Y-L Li L-C Chen W-Q Xu Z-H Bai S-N 2005 Histone acetylation affects expression of cellular patterning genes in the *Arabidopsis* root epidermis. Proceedings of the National Academy of Sciences, USA 102, 14469–14474. 10.1073/pnas.0503143102 PMC1242287 16176989

[CIT0062] Yao X Feng H Yu Y Dong A Shen W-H 2013 SDG2-mediated H3K4 methylation is required for proper *Arabidopsis* root growth and development. PLoS One 8, e56537. 23483879 10.1371/journal.pone.0056537 PMC3585709

