# Supplementary material for: ARABIDOPSIS HOMOLOG of TRITHORAX1 (ATX1) is required for cell production, patterning, and morphogenesis in root development
Source: J Exp Bot. 2014 Sep 9;65(22):6373–84. doi: 10.1093/jxb/eru355 (PMC4246177; doi:10.1093/jxb/eru355)
Supplement: Supplementary Data [file supp_65_22_6373__index.html]

 ARABIDOPSIS HOMOLOG of TRITHORAX1 (ATX1) is required for cell production, patterning, and morphogenesis in root development — ARABIDOPSIS HOMOLOG of TRITHORAX1 (ATX1) is required for cell production, patterning, and morphogenesis in root development — Supplementary Data 

# *ARABIDOPSIS HOMOLOG of TRITHORAX1* (*ATX1*) is required for cell production, patterning, and morphogenesis in root development

## Supplementary Data

Data files

**Files in this Data Supplement:**

- Supplementary Data - Supplementary Data
