# Supplementary material for: ARABIDOPSIS HOMOLOG of TRITHORAX1 (ATX1) is required for cell production, patterning, and morphogenesis in root development
Source: J Exp Bot. 2014 Sep 9;65(22):6373–84. doi: 10.1093/jxb/eru355 (PMC4246177; doi:10.1093/jxb/eru355)
Supplement: Supplementary Data [file supp_eru355_jexbot125583_file001.pdf]

ARABIDOPSIS HOMOLOG of TRITHORAX1 (_ATX1_) is required for cell production, patterning, and morphogenesis in root development

Selene Napsucialy-Mendivil, Raúl Alvarez-Venegas, Svetlana Shishkova, and Joseph G. Dubrovsky

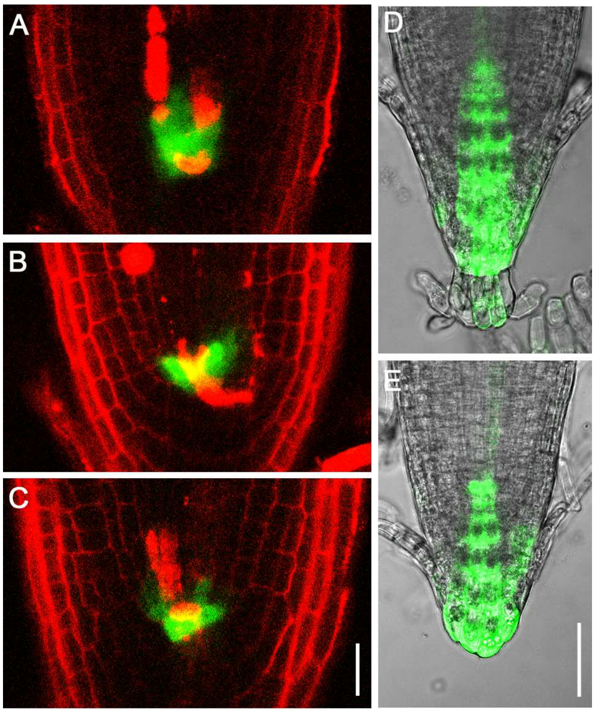

Figure S1. Expansion of the *pWOX5::GFP* expression domain and the auxin response in the primary root apical meristem of the *atx1-1* mutant. (A-C) Root apical meristem (RAM) of *atx1-1* seedlings, *n* = 29. (D and E) *DR5rev::GFP* expression in Wt (Ws) (D) and *atx1-1* (E) primary roots; *n* = 12-15 in three independent experiments; 8 dpg plants were analyzed. Bar = 50 µm.

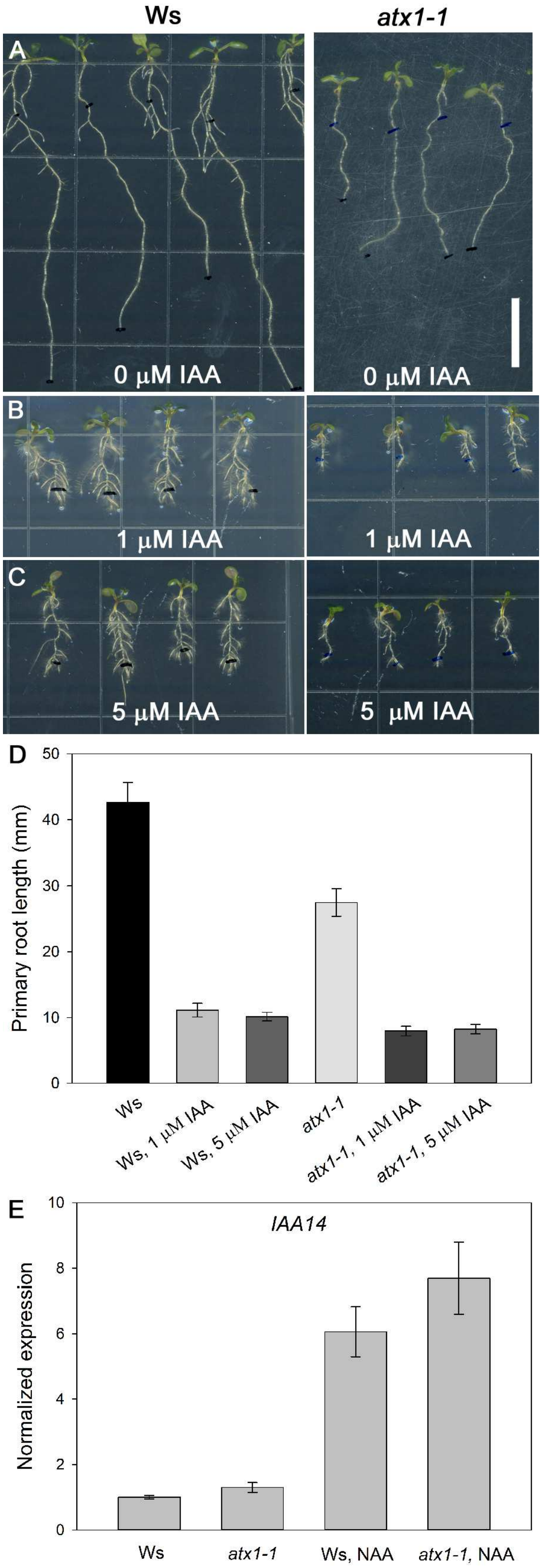

Figure S2. Effect of auxin on root development in Wt (Ws) and *atx1-1* plants. (A) Ws and *atx1-1* seedlings at 8 dpg. (B, C) Seedlings were germinated and grown for 3 days in normal medium and then transferred to the same medium or media supplemented with 1 or 5 µM IAA and grown for five additional days. (D) Primary root length of untreated and IAA-treated seedlings at 8 dpg, *n* = 8. (E) Expression analysis of *IAA14* by RT-qPCR. Plant material was harvested from the roots of 8-dpg Ws and *atx1-1* seedlings untreated or treated with 1 µM NAA for 12 hours. Data were normalized for the expression level of two reference genes, *UBQ10* and *EF1α*. Means ± SE of two independent experiments are shown. Scale bar = 10 mm.
